# Amodiaquine resistance in *Plasmodium berghei* is associated with *PbCRT* His95Pro mutation, loss of chloroquine, artemisinin and primaquine sensitivity, and high transcript levels of key transporters

**DOI:** 10.12688/wellcomeopenres.11768.2

**Published:** 2018-06-06

**Authors:** Loise Ndung'u, Benard Langat, Esther Magiri, Joseph Ng'ang'a, Beatrice Irungu, Alexis Nzila, Daniel Kiboi

**Affiliations:** 1 PAUSTI, Jomo Kenyatta University of Agriculture and Technology, Nairobi, 00200, Kenya; 2 KEMRI- Centre for Traditional Medicine and Drug Research, Kenya Medical Research Institute (KEMRI), Nairobi, 00200, Kenya; 3 Department of Nursing and Nutritional Sciences, University of Kabianga, Kericho, 20200, Kenya; 4 Department of Biochemistry, Jomo Kenyatta University of Agriculture and Technology, Nairobi, 00200, Kenya; 5 Department of Life Sciences, King Fahd University of Petroleum and Minerals, Dharan, 31261, Saudi Arabia; 6 West Africa Centre for Cell Biology and Infectious Pathogens, University of Ghana, Accra, 54 Legon, Ghana; 7 Kenya Medical Research Institute (KEMRI)/Wellcome Trust, Collaborative Research Program, Kilifi, 80108, Kenya

**Keywords:** Malaria, Resistance, Plasmodium berghei, Amodiaquine, Cross-resistance

## Abstract

**Background:** The human malaria parasite *Plasmodium falciparum* has evolved drug evasion mechanisms to all available antimalarials. The combination of amodiaquine-artesunate is among the drug of choice for treatment of uncomplicated malaria. In this combination, a short-acting, artesunate is partnered with long-acting, amodiaquine for which resistance may emerge rapidly especially in high transmission settings. Here, we used a rodent malaria parasite *Plasmodium berghei *ANKA as a surrogate of *P. falciparum* to investigate the mechanisms of amodiaquine resistance.

**Methods**: We used the ramp up approach to select amodiaquine resistance. We then employed the 4-Day Suppressive Test to measure the resistance level and determine the cross-resistance profiles. Finally, we genotyped the resistant parasite by PCR amplification, sequencing and relative quantitation of mRNA transcript of targeted genes.

**Results:** Submission of the parasite to amodiaquine pressure yielded resistant line within thirty-six passages. The effective doses that reduced 90% of parasitaemia (ED _90_) of the sensitive and resistant lines were 4.29mg/kg and 19.13mg/kg respectively. The selected parasite retained resistance after ten passage cycles in the absence of the drug and freezing at -80ºC for one month with ED _90_ of 20.34mg/kg and 18.22mg/kg. The parasite lost susceptibility to chloroquine by (6-fold), artemether (10-fold), primaquine (5-fold), piperaquine (2-fold) and lumefantrine (3-fold). Sequence analysis of *Plasmodium berghei chloroquine-resistant transporter* revealed His95Pro mutation. We found no variation in the nucleotide sequences *of Plasmodium berghei multidrug resistance gene-1 (Pbmdr1), Plasmodium berghei deubiquitinating enzyme-1* or *Plasmodium berghei Kelch13 domain*. However, high mRNA transcripts of essential transporters; *Pbmdr1*, V-type/H+ pumping pyrophosphatase-2 and sodium hydrogen ion exchanger-1 and Ca ^2+^/H ^+^ antiporter accompanies amodiaquine resistance.

**Conclusions:** The selection of amodiaquine resistance yielded stable “multidrug-resistant’’ parasites and thus may be used to study shared resistance mechanisms associated with other antimalarial drugs. Genome-wide analysis of the parasite may elucidate other functionally relevant genes controlling AQ resistance in *P. berghei*.

## Introduction

The malaria parasite *Plasmodium falciparum* causes the highest disease burden and death in developing countries. In 2015, the World Health Organization reported 200 million clinical malaria cases with 400,000 cases resulting in death ( WHO, 2016). The majority of this burden is in sub-Saharan Africa, primarily in children under five years of age. With the newly introduced vaccine showing less than 50% reduction in the clinical cases and its efficacy waning with time ( Olotu *et al.*, 2013; RTS,S Clinical Trials Partnership, 2014), the use of drugs for prevention and treatment of malaria remains an essential alternative in malaria control. To date, the treatment of uncomplicated malaria relies on the artemisinin-based combination therapies (ACTs), comprising of the short-acting artemisinin derivative and a long-acting partner drug, a strategy intended to reduce the emergence of resistance ( WHO, 2016). However, the genetically flexible malaria parasite has evolved drug evasion mechanisms to all available antimalarial drugs, including the artemisinins ( Amaratunga *et al.*, 2016; Amato *et al.*, 2017; Miotto *et al.*, 2015).

The ACTs are currently used widely in many African countries where malaria is endemic; however, the extensive use is against the backdrop of high malaria transmissions, exposing the long-acting partner drugs to intense selection pressures ( White, 2002). For instance, the combination of amodiaquine and artesunate (AQ-ASN) is among the five recommended ACTs for treatment of uncomplicated malaria ( WHO, 2016). This combination is available as a fixed combination Coarsucam™/Winthop®, Sanofi-Aventis ( Gil, 2008). The ASN is a short-acting drug with a half-life of <2hours ( Robert *et al.*, 2001; Tilley *et al.*, 2016). On the other hand, AQ is a prodrug that is rapidly metabolised to its active long-acting metabolite desethylamodiaquine (DEAQ), with a half-life of more than five days ( Churchill *et al.*, 1985). In some African countries, AQ-ASN is the first or a second line drug for treatment of uncomplicated malaria ( Rwagacondo *et al.*, 2004; Sondo *et al.*, 2016; WHO, 2016). In areas of highly seasonal transmission, such as sub-Sahel region, the AQ and sulfadoxine/pyrimethamine (AQ-SP) is used as a prophylactic combination, in children below five years, of age ( WHO, 2016). Thus, AQ remains a useful drug in the treatment and prevention of malaria infection.

Amodiaquine like chloroquine (CQ) belongs to 4-amino-quinolines class of the antimalarial drugs, and their mechanisms of resistance are predicted to be similar. However, AQ is active against some CQ resistant parasite strains ( Basco & Ringwald, 2003; Gorka *et al.*, 2013; Sa *et al.*, 2009), suggesting that the mechanisms of resistance may be different. The resistance to 4-amino-quinoline drugs in *Plasmodium falciparum* strongly associate with polymorphisms in two essential genes. First, *Plasmodium falciparum chloroquine resistance transporter* ( *Pfcrt*) Lys76Thr change is associated with CQ resistance and decreased sensitivity to AQ ( Ecker *et al.*, 2012; Fidock *et al.*, 2000; Ochong *et al.*, 2003). Second, in the presence of *Pfcrt* Lys76Thr mutation, *Plasmodium falciparum multidrug resistance gene 1* ( *Pfmdr1*), Asn86Tyr mutation enhances CQ resistance and decreases AQ sensitivity ( Ferdig *et al.*, 2004; Fidock *et al.*, 2000; Holmgren *et al.*, 2006; Wellems, 2002). Currently, the mechanisms of AQ resistance are poorly understood. To extensively study these mechanisms, one needs to obtain naturally occurring stable *P. falciparum* lines resistant to AQ, but such parasites are not available. This limitation is overcome by inducing resistance *in vitro* using *P. falciparum* or *in vivo* using murine malaria parasites. However, exposing drug-sensitive *P. falciparum* parasite to drug concentrations to select stable-drug-resistant lines is a cumbersome and time-consuming process ( Nzila & Mwai, 2010). On the other hand, stable-resistant parasites lines can be induced *in vivo,* with relative ease, using a rodent model in mice, and these rodent parasites can be used as a surrogate of *P. falciparum* to study the mechanisms of drug resistance ( Carlton *et al.*, 2001). Although some drug resistance mechanisms between *P. falciparum* and murine malaria do not correlate ( Afonso *et al.*, 2006; Carlton *et al.*, 2001; Hunt *et al.*, 2007), other mechanisms are similar. For instance, mefloquine (MQ) resistant *P. berghei* lines ( Gervais *et al.*, 1999) demonstrated overexpression on *the mdr1* gene, the gene associated with MQ resistance in *P. falciparum*, *P. berghei* and *P. chabaudi* ( Cravo *et al.*, 2003; Price *et al.*, 2004) *.* Similarly, non-synonymous mutations in *the cytochrome b* gene associates with atovaquone resistance in *P. berghei*, *P. chabaudi* and *P. falciparum* ( Afonso *et al.*, 2006; Srivastava *et al.*, 1999; Syafruddin *et al.,* 1999). Mutations in the dihydrofolate reductase ( *dhfr*) and dihydropteroate synthase ( *dhps*) genes are associated with sulphadoxine and pyrimethamine resistance in *P. chabaudi* and *P. falciparum* ( Culleton *et al.*, 2005; Martinelli *et al.*, 2011). These studies support the utility of murine malaria as surrogate models for identifying drug resistance genes in *P. falciparum*.

In this study, we report on the *in vivo* selection of stable AQ resistant murine malaria *Plasmodium berghei* ANKA parasite lines, and their use in investigating the mechanisms of AQ resistance. As discussed earlier, AQ and CQ are quinoline-based drugs and resistance to CQ is associated with the decreased susceptibility to AQ. Some markers of resistance to other quinoline drugs, such as lumefantrine (LM), piperaquine (PQ) and quinine (QN) modulate the susceptibility to CQ ( Eastman *et al.*, 2011; Mwai *et al.*, 2012; Okombo *et al.*, 2010; Witkowski *et al.*, 2017). Since all these drugs are proffered to have common mechanisms of action, which is the inhibition of heme detoxification ( Muller & Hyde, 2010; O’Neill *et al.*, 2006; Robert *et al.*, 2001). We hypothesised that selected resistance markers associated with the quinoline drugs mentioned above also modulate parasite susceptibility to AQ. These markers in addition to *Pfcrt* and *Pfmdr1*, are the deubiquitinating enzyme 1 ( *ubp1*), which is linked with resistance to CQ and artesunate in *Plasmodium chabaudi* ( Hunt *et al.*, 2007; Hunt *et al.*, 2010), and artemisinin tolerance in *P. falciparum* ( Henriques *et al.*, 2014) *.* The V-type H+ pumping pyrophosphatase 2 ( *vp2*) and Ca ^2+^/H ^+^ antiporter ( *vcx1*) which modulate resistance to CQ, LM and PQ in *P. falciparum* and *P. berghei* ( Gonzales *et al.*, 2008; Kiboi *et al.*, 2014). Also, the *P. falciparum sodium-hydrogen ion exchanger 1* ( *Pfnhe1*), which modifies pH gradient between the digestive vacuole and cytosol milieu and regulates quinine resistance in *P. falciparum* ( Bennett *et al.*, 2007) *.* Thus, using the selected stable AQ resistant parasite line, we assessed for the presence of synonymous SNP and measured the transcript levels of *the* markers mentioned above in AQ resistant P. berghei parasites. Finally, the role of the *Kelch13* propeller, a protein domain involved in detecting intracellular oxidative stress resulting from artemisinin and other endoperoxides action and a marker for artemisinin resistance in *P. falciparum* ( Leroy, 2017; Miotto *et al.*, 2015; Straimer *et al.*, 2015) was also studied.

## Materials and methods

### Parasites, host and compounds

Male Swiss albino mice (6–7 weeks old) weighing 20±2g outbred at KEMRI Animal House (Nairobi, Kenya) were used to induce AQ resistance from sensitive parasite line of *P. berghei* ANKA (MRA-868, MR4, ATCC® Manassas, Virginia, 676m1cl1). The animals were kept in the animal house in standard polypropylene cages and fed on commercial rodent feed and water *ad libitum*. AQ, CQ, primaquine (PMQ) **,** LM, artemether (ATM) and PQ) were prepared freshly by dissolving in a solvent containing 3% ethanol and 7% Tween-80. In all mouse experiments, at least three mice were used per experimental group to allow the calculation of averages, standard deviation and statistical analysis.

### Determination of 50% and 90% effective doses

The 50% and 90% effective doses that reduce parasitaemia by 50% (ED _50_) and 90% (ED _90_) respectively, after four consecutive drug dosages were determined following quantitative standard 4-Day Suppressive Test (4DT) ( Fidock *et al.*, 2004). Briefly, twenty-five mice were randomly infected intraperitoneally each with 1×10 ^6^ parasites and then randomly allocated to the four test groups and the control group (five mice per group). Oral treatment with the drug started on day 0, (2–4 hrs post-infection) and continued for four days, days 0–3 (24, 48 and 72 hrs post-infection). Parasite density for ED _50_ and ED _90_ calculation was estimated microscopically (×100) on day 4 (96 hrs) post parasite inoculation using thin blood films made from tail blood snips. The parasite growth was monitored on D2, D3, D4, D7, D9, D11 and D15 days post infection. Percentage chemo-suppression of each dose was calculated following the formula ( Fidock *et al.*, 2004). The ED _50_ and ED _90_ were then estimated using linear regression line.

### Submission of the parasite to AQ pressure and testing the resistance levels

The AQ sensitive parasites were submitted to continuous AQ pressure. At least six mice (three for the control and three for the test group) were inoculated intraperitoneally each with 1×10 ^6^ parasitised red blood cells in a 0.2ml on day 0 (D0). The parasitaemia was then allowed to rise >5% when test mice were treated orally with AQ at a concentration equivalent to the ED _99_. The parasite growth was then monitored to between 2–7% when donor mice were selected for subsequent passage into the next naive group of three mice. The parasites were then exposed to an increasing concentration of AQ in the subsequent passages based on parasite growth. The level of acquired resistance was evaluated at an interval of four drug pressure passages by measuring the ED _50_ and ED _90_ in the standard 4DT. Two approaches were employed to confirm the stability of the acquired resistance; first by freezing the selected AQ resistant parasite at -80°C for at least one month, second the AQ resistant parasites were passaged for at least ten passages in the absence of the drug. The ED _50 _and ED _90_ values were determined after the freezing-thawing process and after the ten mechanical passages in the absence of the drug. The ED _90_ allowed us to calculate the 90% index of resistance (I _90_) from the ratio of the ED _90_ of the resistant line to that of sensitive parent line. Based on I _90_ value, resistance levels were classified into four categories: i) I _90_ **=**1.0 (sensitive), ii) I _90 _= 1.01-10.0 (slightly resistance), iii) I _90_=10.01-100, (moderate resistance), iv) I _90 _≥100 (high resistance) ( Xiao *et al.*, 2004).

### Generation of the genetically homogeneous parasite by dilution cloning

During the selection of resistant lines using the ramp up approach, a high parasite density of approximately 1×10 ^6^ infected red blood cells is submitted to the increasing drug pressure. Consequently, the parasites accumulate mutations. To minimise the random variation occurring during the selection process, we generated a genetically homogenous clone using the limiting dilution approach, as detailed by ( Janse *et al.*, 2004). Briefly, a mouse with parasitaemia between 0.5 and 1% was selected as a donor mouse. Five microlitres of infected blood were collected from the tail snip of the mouse in 1µl of heparin and diluted in 1ml of 1× PBS. The number of infected erythrocytes per 1µl was estimated from 20µl of the diluted blood. The cell suspension was then diluted further with 1×PBS to an estimated final concentration of 0.5 parasites/ 0.2ml PBS. 12 mice were then intravenously injected with the infected blood. Cloning was considered successful when 3 to 6 mice had a parasitaemia of between 0.3–0.5% at day eight post-infection. The fastest growing clone was selected for the subsequent cross-resistance and molecular studies.

### Evaluation of cross-resistance profiles

The sensitivity of the selected AQ-resistant parasites line against other antimalarial drugs, DEAQ, CQ, PMQ, PQ, ATM and LM, was also investigated by measuring the ED _50_ and ED _90_ in the 4DT assay ( Fidock *et al.*, 2004). The ED _50_ and ED _90 _of the resistant parasite were compared to the ED _50_ and ED _90_ of sensitive parental line. To this purpose, four different drug concentrations were selected for each of the test drugs and administered orally, except for DEAQ which was administered intraperitoneally. The 50% and 90% indices of resistance were calculated as previously discussed.

### DNA extraction, PCR and sequencing of *Pbmdr1*, *Pbcrt*, *Pbubp1* and *PbKelch13*

**Figure 1.**
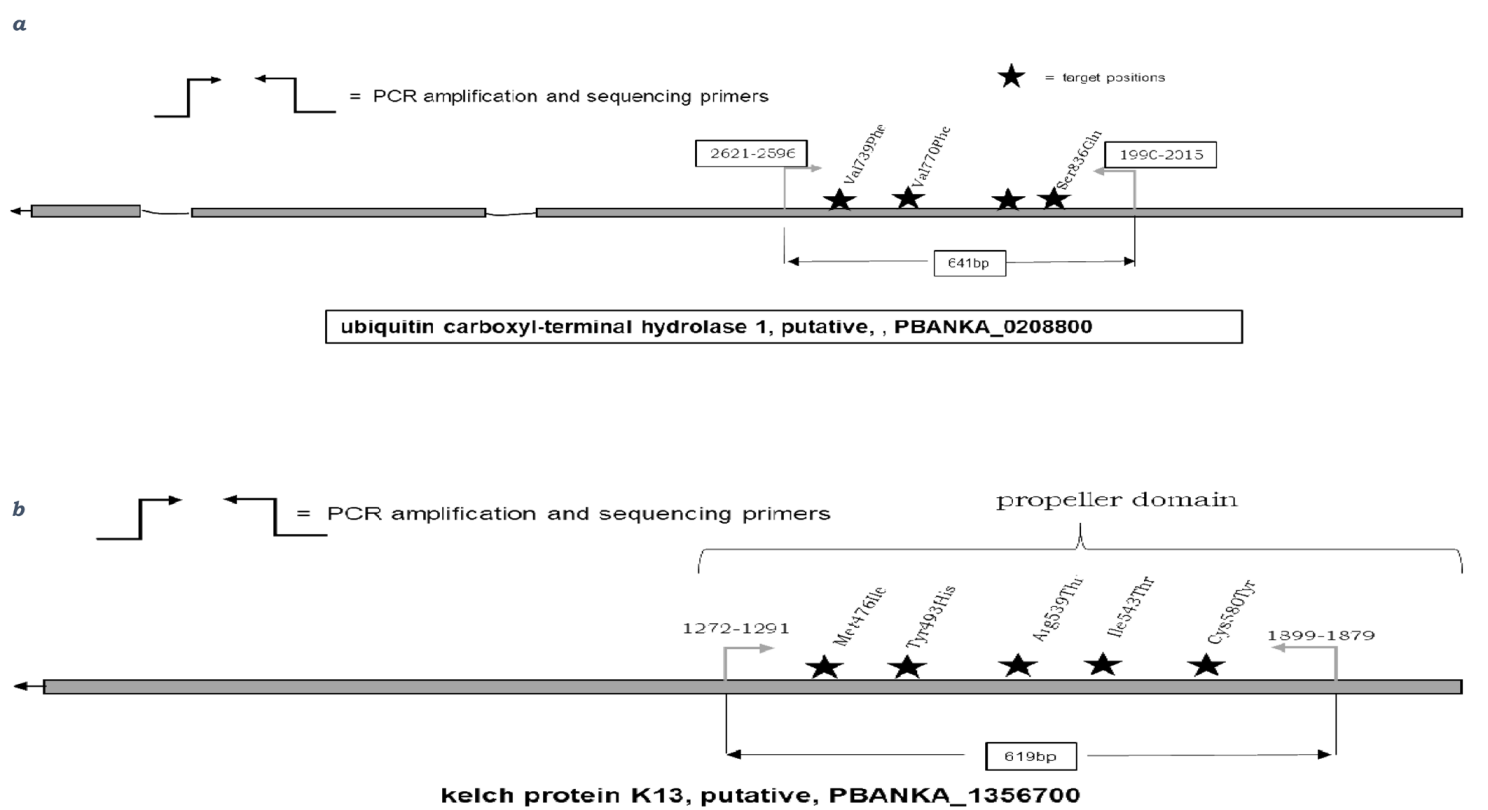
Genome view of drug resistance genes and target regions. ( **a**) *Plasmodium berghei* ubiquitin carboxyl-terminal hydrolase 1, and ( **b**) *Plasmodium berghei kelch 13* protein, putative showing targeted positions (*), annealing positions for PCR and sequencing primers and the sizes of amplified PCR products.

Evaluation for the presence of SNPs in *Pbmdr1*, *Pbcrt*, *Pbubp1* and *PbKelch13 genes* was carried out by sequencing, after PCR amplification from genomic DNA (gDNA) or cDNA generated from the mRNA. As illustrated in Figure 1a–b, target fragments corresponding to specific regions of interest from the *Pbubp1* (PBANKA_0208800) and *PbKelch13* (PBANKA_1356700) were PCR amplified from gDNA and sequenced using primers commercially synthesised from Inqaba Biotechnical Industries (Pty) Ltd, South Africa. The whole coding regions of the *Pbcrt* (PBANKA_1219500) and *Pbmdr1* (PBANKA_1237800) genes were amplified from the cDNA or gDNA template using primers listed in Table 1a. In extracting parasite genomic DNA (gDNA), 500µl of infected mouse blood with 5–10% parasitaemia was diluted with 500µl of 1×PBS, and the solution spun for 1 min at 500×g. After aspiration of the supernatant, the pellet was resuspended in a 30ml volume of cold 4°C 1×erythrocytes lysis buffer for 30 minutes, followed by spinning at 500×g for 10 min. The parasite pellet was washed twice with 30ml 1×PBS with centrifugation at 500×g for 5 min at 4°C. Genomic DNA (gDNA) was extracted using a commercial QIAamp® Blood DNA extraction kit (Qiagen) following the manufacturer’s instructions. For the PCR amplification, 1µl of gDNA was used as the template in 25µl PCR reactions using the DreamTaq Master Mix or Phusion Flash High Fidelity Master Mix (Thermo-Scientific™). Table 1b shows the optimised cycling conditions. The PCR products were first analysed in 1.5% agarose gel, purified using the GeneJet™ PCR purification kit (Thermo Scientific™) and then sequenced using a 3730xlsequencer based on BigDye v3.1. DNA sequences were analysed using Lasergene 11 Core Suite and CLUSTAL Omega ( http://www.ebi.ac.uk/Tools/msa/clustalo/) and PlasmoDB ( http://plasmodb.org/plasmo/) ( PlasmoDB, 2017).

**Table 1.** PCR methods. ( **A**) Primer sequences for the PCR amplification and sequencing of *Plasmodium berghei* chloroquine resistance transporter ( *Pbcrt*), *Plasmodium berghei* multidrug resistance gene 1 ( *Pbmdr1*), *Plasmodium berghei* ubiquitin carboxyl-terminal hydrolase 1 ( *Pbubp1*) and *Plasmodium berghei* kelch 13 protein, putative ( *Pbkelch13*) genes ( **B**) Optimized condition for PCR amplification *Plasmodium berghei* chloroquine resistance transporter ( *Pbcrt*), *Plasmodium berghei* multidrug resistance gene 1 ( *Pbmdr1*), *Plasmodium berghei* ubiquitin carboxyl-terminal hydrolase 1 ( *Pbubp1*) and *Plasmodium berghei* kelch 13 protein, putative ( *Pbkelch13*) genes.

TABLE 1A				
Primer Name	PCR primers sequence (5' to 3')		Primer annealing position	
*Pbcrt - Forward*	GGA CAG CCT AAT AAC CAA TGG		69-89	
*Pbcrt - Reverse*	GTT AAT TCT GCT TCG GAG TCA TTG		1230-1253	
**Sequencing primers (5' to 3')**				
*Pbcrt - Forward*	GGA CAG CCT AAT AAC CAA TGG		69-89	
*Pbcrt - Reverse*	CGA CCA TAG CAT TCA ATC TTA GG		751-729	
*Pbcrt - Forward*	TCA GGA AGA AGT TGT GTC A		109-127	
*Pbcrt - Reverse*	GAT AAG GAA AAA CTG CCA TC		383-402	
*Pbcrt - Forward*	GTG TTG GCA TGG TCA AAA TG		908-927	
*Pbcrt - Reverse*	CTT GGT TTT CTT ACA GCA TCG		1124-1104	
*Pbcrt - Forward*	CCT AAG ATT GAA TGC TAT GGT CGT		729-751	
*Pbcrt - Reverse*	GTT AAT TCT GCT TCG GAG TCA TTG		1230-1253	
**PCR primers (5' to 3')**				
*Pbkelch13 - Forward*	AGT CAA ACA GTA TCT CTA ACT		20-40	
*Pbkelch13 - Reverse*	ACG GAA TGT CCA AAT CTT G		2198-2180	
**Sequencing primers (5' to 3')**				
*Pbkelch13 - Forward*	TCC ACT AAC CAT ACC TAT AC		1272-1291	
*Pbkelch13 - Reverse*	AGC TTC TAA TAA TGC ATA TGG		1899-1879	
**PCR primers (5' to 3')**				
*Pbmdr1 - Forward*	GTCTAAATGTTGTAATTTGTTGTCCT		196bp upstream	
*Pbmdr1 - Reverse*	GACATTATCTAATTTCATCACCTTG		180bp downstream	
**Sequencing primers (5' to 3')**				
*Pbmdr1 - Reverse*	CAGTATCATTCACACTTTCTCC		250-271	
*Pbmdr1 - Forward*	GTGCAACTATATCAGGAGCTTCG		176-198	
*Pbmdr1 - Reverse*	CACTTTCTCCACAATAACTTGCTACA		717-742	
*Pbmdr1 - Forward*	GCAGCTCTATATGTAATAAAAGGGTC		611-636	
*Pbmdr1 - Reverse*	GTCGACAGCTGGTTTTCTG		1062-1080	
*Pbmdr1 - Forward*	CTTTGAATTACGGTAGTGGCT		908-928	
*Pbmdr1 - Reverse*	TCGCTAGTTGTATTCCTCTTAGA		1549-1571	
*Pbmdr1 - Forward*	TGGAGTAGTTAGTCAAGATCCT		1362-1383	
*Pbmdr1 - Reverse*	GTGCCTTGTTCAACTATTACAC		2000-2021	
*Pbmdr1 - Forward*	TCAAATAGAGATCAAGAATCAACAGG		1930-1955	
*Pbmdr1 - Reverse*	GGATATAAACCACCTGCCACT		2379-2399	
*Pbmdr1 - Forward*	GCCAAGTAAACCATCATTCTTCA		2247-2269	
*Pbmdr1 - Reverse*	TCGCGTTGTAATGGTATATGCT		2803-2823	
*Pbmdr1 - Forward*	GGATTTTTATCGTCGCATATTAACAG		2647-2672	
*Pbmdr1 - Reverse*	TAGCTTTATCTGCATCTCCTTTGAAG		3234-3259	
*Pbmdr1 - Forward*	TGCAATAGATTATGACAGTAAAGGGG		3021-3046	
*Pbmdr1 - Reverse*	ATCTTTCAAATCGTAGAATCGCAT		3513-3538	
*Pbmdr1 - Forward*	CTTCAAAGGAGATGCAGATAAAGCTA		3234-3259	
*Pbmdr1 - Reverse*	GATTCAATAAATTCGTCAATAGCAGC		3862-3887	
*Pbmdr1 - Forward*	TGCAATAGTTAACCAAGAACCAATGT		3753-3778	
*Pbmdr1 - Reverse*	TAGATGCAATTCTGTGAGCAATAG		4100-4123	
**PCR and Sequencing primers (5' to 3')**				
*Pbubp1 - Forward*	AGT TCC AAT GAA TAT ATT CAT GTG AA		1990-2015	
*Pbubp1 - Reverse*	CTA AGT TGC ATA GCT TTA TCA TTT TC		2621-2596	
TABLE 1B				
PCR amplifying profiles	Temperature (°C)/Time (min)			
	*Pbcrt*	*Pbmdr1*	*Pbubp1*	*Pbkelch13*
Initial denaturation	98°C, 30 secs	98°C, 30 secs	95°C, 5 min	95°C, 5 min
Denaturation	98°C, 10 secs	98°C, 10 secs	95°C, 30 secs	95°C, 1 min
Annealing Temperature	50°C, 15 secs	52°C, 15 secs	50°C, 30 secs	51°C, 30 secs
Elongation	72°C, 30 secs	72°C, 1 min	72°C, 1.5 min	72°C, 1.5 min
Primer (Forward & reverse)	2.5µM each	2.5µM each	2.5µM each	2.5µM each
MgCl2 (mM)			1.5	2.0
dNTPs (mM)			2.0	2.0
Cycles	30	30	30	30
Final elongation	72°C, 2 min	72°C, 2 min	72°C, 10 min	72°C, 10 min

### RNA extraction, cDNA synthesis and qRT-PCR assays

The quantity of the mRNA transcripts of *Pbmdr1*, *Pbvp2*, *Pbvcx1,* and *Pbnhe1* genes was carried out after cDNA synthesis from mRNA. Before the extraction of RNA, all the buffers and solutions for parasite preparation were treated with 0.1% (v/v) of diethyl pyrocarbonate (DEPC). The total RNA was isolated from approximately 1×10 ^6^ fresh parasites pellet. In preparation of parasite pellet, parasitised red blood cells were first washed in 1×PBS and then lysed in 5 volumes of ammonium chloride solution. The parasite pellet was washed twice in 10ml of 1×PBS and then resuspended in 200µl of 1×PBS. Total RNA was isolated using Quick-RNA™ MiniPrep (Zymo Research™) following the manufacturer’s instructions. The first strand cDNA synthesis was performed in a final volume of 20µl using RevertAid First Strand cDNA synthesis kit and oligo-DT as primers. Five micrograms of the total RNA, 1µl of oligo-DT and water were mixed with 4µl Reaction buffer (5×), 1µl RiboLock RNase Inhibitor (U/µl), 2µl of dNTPs (10mM) and 1µl of RevertAid M-MuLV RT (200U/µl). The reaction mix was first incubated at 42°C for 60min, then at 70°C for 5min and finally chilled on ice. The cDNA was used as the template for qRT-PCR assays.

The mRNA transcript levels were evaluated using qRT-PCR in a final volume of 20µl using Maxima SYBR Green/ROX qPCR Master Mix (Thermo Scientific™). Oligonucleotide for *Pbmdr1*, *Pbvp2*, *Pbvcx1* and *Pbnhe1* were designed to run using similar cycling conditions relative to the *Pbβ-actin I*, as the housekeeping gene ( Table 2). Briefly, 12µl of Maxima SYBR mix, 2.0µl (0.25µM) of forward and reverse primers each, 1µl cDNA and 3µl water were mixed. The reaction mix was run for pre-treatment at 50°C, for 2 min; initial denaturation at 95°C for 10 min; denaturation at 95°C for 15 secs; and annealing at 60°C for 60 secs for 45 cycles.

**Table 2.** Oligonucleotide sequences used in the q PCR assays. The oligos were utilised to measure the transcriptional level profiles of *Plasmodium berghei* multidrug resistance gene 1 ( *Pbmdr1), Plasmodium berghei* V-type H+ pumping pyrophosphatase ( *Pbvp2), Plasmodium berghei* Ca ^2+/^H ^+^ antiporter ( *Pbvcx1), Plasmodium berghei* sodium hydrogen exchanger *(Pbnhe1) genes with Plasmodium berghei β-actin I gene (Pbβ-actin I) as housekeeping using Maxima SYBR Green chemistry in qPC* *R*.

Name	Primer sequence (5’ - 3’)	Position	Tm
*Pbmdr1 -* Forward	ACGGTAGTGGCTTCAATGGA	917-936	54.2
*Pbmdr1 -* Reverse	CTGTCGACAGCTGGTTTTCTG	1082-1062	54.7
*Pbnhe1 -* Forward	TGGAGAGTTTGATTTAGGCTTACC	2022-2045	54.0
*Pbnhe1 -* Reverse	GCTAGGCGATGTTTTGTTAGGAG	2202-2180	55.3
*Pbvp2* - Forward	TGCAGCAGGAAATACAACAGC	1449-1469	55.2
*Pbvp2* - Reverse	GTCGTACTTTTGCACTACTTGCGT	1558-1535	56.5
*Pbcvx1* - Forward	TCAAATTGCTCTTTTTGTTGTACCAA	1101-1126	57.9
*Pbcvx1 -* Reverse	ACACCTTCTAGCCAATTACTTTCACC	1265-1240	57.1
*Pbβ-actin I* - Forward	CAGCAATGTATGTAGCAATTCAAGC	392-416	56.8
*Pbβ-actin I* - Reverse	CATGGGGTAATGCATATCCTTCATAA	523-498	58.9

### Statistical analysis

The means of expression levels of each gene from three independent experiments and from triplicate assays obtained from AQ resistant were compared to AQ sensitive using Student’s t-test; p-value was set at 0.05. The relative expression level results were normalized using *Pbβ-actin I* as the housekeeping using the formula 2 ^ΔΔ^CT based on Livak & Schmittgen, 2001. The means for cross-resistance profiles for each drug from at least four different drug concentrations were analysed using Student’s t-test, with *p*-value set at 0.05.

### Ethical approval

This study was conducted at KEMRI. All animal work was carried out as per relevant national and international standards, as approved by KEMRI-Animal Use and Care Committee. Permission to carry out this study and ethical clearance was approved by KEMRI’s Scientific Ethics Review Unit (No 3378).

## Results and discussion

### Amodiaquine drug pressure induces stable, resistant phenotypes

**Figure 2.**
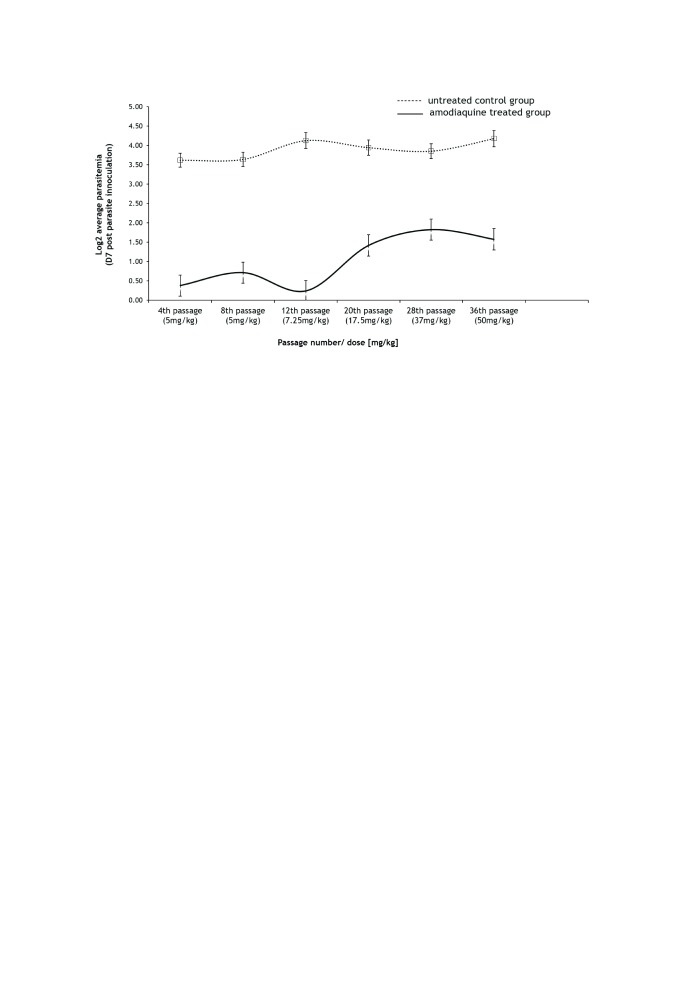
Log2 average parasitaemia of *Plasmodium berghei* ANKA during the selection of amodiaquine resistance. The growth profiles of the parasites from the untreated control group and amodiaquine treated group at the different passage stages and the different drug concentrations during the selection of the amodiaquine resistant parasites.

**Figure 3.**
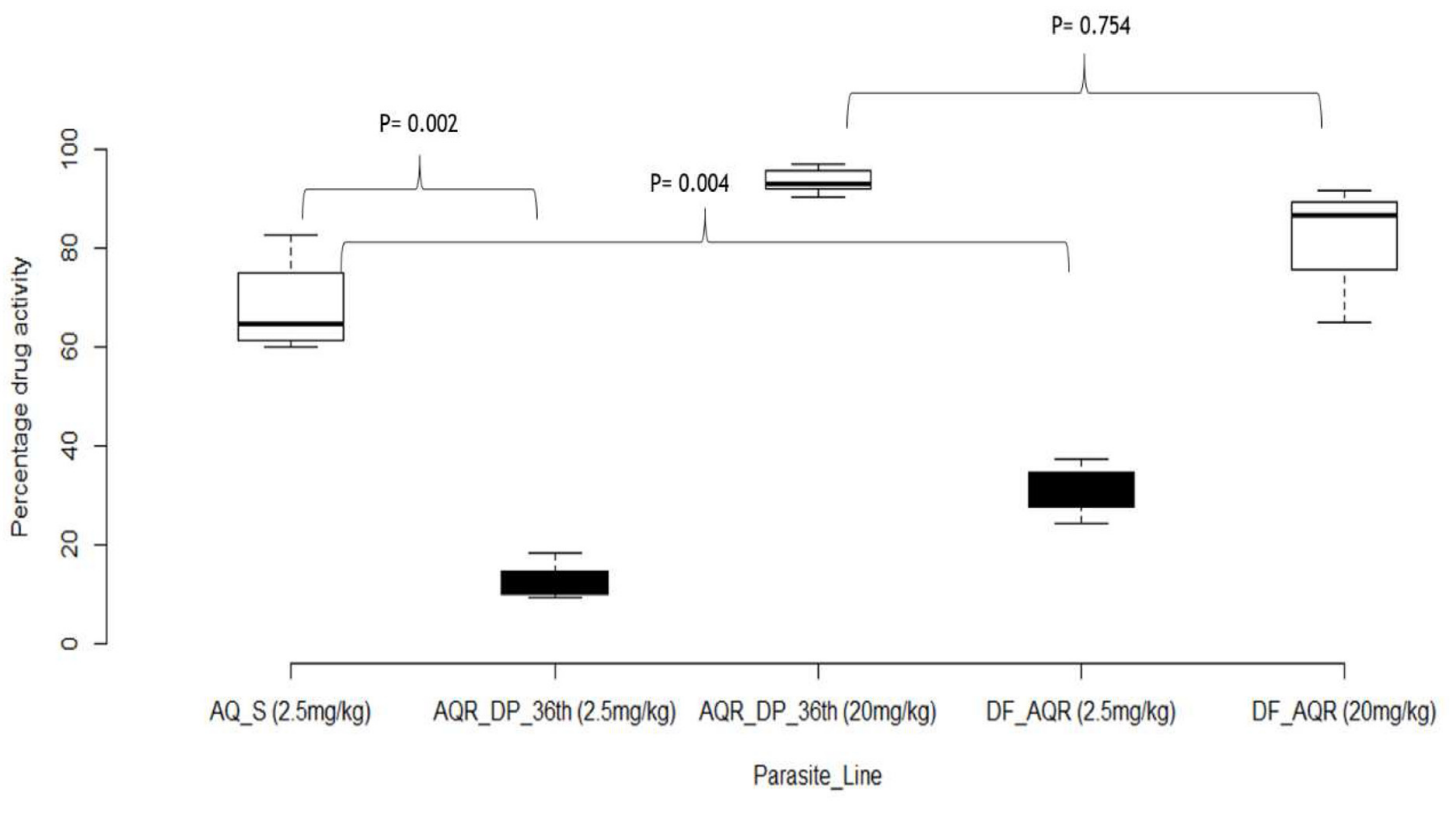
Stability of the amodiaquine resistant line. Percentage activity of the amodiaquine against the drug-sensitive parent line (AQ_S), the 36 ^th^ passage AQ resistant line (AQR_36 ^th^) and the drug-free AQ resistant line (DF_AQR). The AQ_S parasite line remained susceptible to AQ at 2.5mg/kg but both the AQR_36 ^th^ and DF_AQR parasite line retained the resistance level as portrayed by the responses to both 2.5mg/kg and 20mg/kg of AQ. The 90% effective dosage for AQ_S, AQR_36 ^th^ and DF_AQR was 4.29mg/kg, 19.13mg/kg, 20.34mg/kg respectively.

The current introduction of AQ as a component of the ACT therapy ( Gil, 2008) has spurred studies on understanding the mechanisms of AQ resistance. Using the 2% Relapse approach; the AQ resistant *P. berghei* and *P. yoelii* were generated by submitting the parasites to 60mg/kg and 100mg/kg respectively ( Peters & Robinson, 1992); however, the stability, resistance indices and molecular mechanisms remained undetermined. Here we demonstrate that stable AQ resistant *P. berghei ANKA* can be achieved by submitting sensitive parasites to thirty-six continuous drug pressure passages ( Dataset 1). To initiate selection of resistance, we first determined the ED _50_, ED _90_ and ED _99 _of AQ against the sensitive *P. berghei* ANKA. The ED _50_, ED _90_, ED _99 _were 0.95, 4.29 and 5.05mg/kg/day, respectively. We adopted the ramp up approach which employs the sequential increase in the drug pressure. The 5.05mg/kg drug concentration was the starting drug pressure dose and administered once percentage parasitaemia rose to 2–7%. At the onset, average parasitaemia reached 2–7% on day 3–4 post-infection, after which mice received 5.05mg/kg of AQ. Figure 2 shows parasite responses to AQ at the different passages and the different drug concentrations during the selection of drug-resistant parasites. On average, recovery of the parasites from the treated donor mouse was on day seven post-infection. Based on parasite growth at different passages, the drug pressure dose was increased by a factor of ED _99 _at different passage levels. Within the first twelve passages, administration of single 5mg/kg of AQ, after attaining >2% parasitaemia, cleared the parasite to below detectable levels by microscopy. The parasite density of >2% parasitaemia was attained after 7–10 days; therefore, the same drug pressure dose was administered for the first twelve passages. From the 13 ^th^ passage, the parasite recrudescence after drug treatment reduced from 7 days to 3–4 days. We henceforward increased the drug pressure dose by a factor of 1.5 of the ED _99_ (equivalent to 2.5mg/kg) after every two passages up to the 20 ^th^ passage. From the 20th passage, we increased the drug pressure dose sequentially by a factor of 2 of the ED _99_ (equivalent to 5mg/kg) after every two passages. By the 36 ^th^ passage, the drug pressure dose had risen to 50mg/kg. The 50mg/kg dose was fifty and ten times higher than the ED _50_ and ED _99_ of the parent line respectively. When we quantified the ED _50_ and ED _99_ in the 4DT, we expected higher indices of resistance. Surprisingly the I _50_ and I _90_ were only twelve and four folds respectively ( Table 3a). The resistant line remained stable after freezing at -80°C for at least one month, with ED _50 _and ED _90_ of 5.86mg/kg and 18.22mg/kg respectively. Similarly, the ED _50_ and ED _90 _values after ten drug-free passages corresponded to 8.05mg/kg and 20.34mg/kg respectively ( Table 3a). We then tested drug response of the 36 ^th ^passage AQ resistant line (AQR_36 ^th^), drug-sensitive parent line (AQ_S), and drug-free AQ resistant line (DF_AQR) at 2.5mg/kg and 20mg/kg of AQ. As expected, 2.5mg/kg was active against the AQ_S with 68%. However, the same concentration yielded a mere 12.5% and 31% activity against the AQR_36 ^th ^and DF_AQR respectively ( Figure 3). On increasing the drug concentration to 20mg/kg, we recorded a 96% and 83% activity against the AQR_36 ^th ^and DF_AQR. Our data indicate that the AQR parasite line retained an index of resistance after the ten passages in the absence of the drug and freeze-thawing process. We thus concluded that stable-AQ resistant *P. berghei* parasite line was successfully selected and the resistance mechanisms are probably encoded in the cell genome.

**Table 3.** Amodiaquine resistance and cross-resistance levels. ( **A**) The 50% and 90% Effective Dose (ED _50_ and ED _90_) in mg/kg/day of amodiaquine resistant Plasmodium berghei ANKA line at different passage levels showing a sharp rise in ED _50_ in comparison to the steady but slow increase in ED _90_. Index of resistance at 50% (I _50_) and 90% (I _90_) from the ratio of ED _50_ or ED _90_ of the resistant line with ED _50_ or ED _90_ of sensitive line respectively. The effective dose was measured in the 4-Day suppressive Test using at least four different drug concentrations and at least four Swiss mice per dose. ( **B**) Cross-resistance profiles of the amodiaquine resistant *Plasmodium berghei* ANKA line and sensitive parent line as measured in the 4-Day suppressive Test using at least four different drug concentrations and at least four Swiss mice per drug concentration. The Index of resistance (I _90_) calculated from the ratio of ED _90_ of the resistant line to that of the sensitive parent line.

TABLE 3A				
Passages No.	50% and 90% effective dose		Index of resistance	
	ED _50_	ED _90_	I _50_	I _90_
1 ^st^	0.95	4.29	1.00	1.00
4 ^th^	1.07	3.59	1.13	0.84
8 ^th^	1.90	4.06	2.00	0.95
12 ^th^	2.26	4.13	2.38	0.96
20 ^th^	2.63	4.55	2.76	1.06
28 ^th^	5.00	11.44	5.26	2.67
36 ^th^	12.01	19.13	12.64	4.46
Stability after freezing for one month	5.86	18.22	6.17	4.24
Stability results after ten passages in the absence of the drug	8.05	20.34	8.47	4.74
TABLE 3B				
Antimalarial drug	Sensitive parental line	Amodiaquine resistant line		Index of resistance
	ED _90_	ED _90_		I _90_
Primaquine	1.74	7.76 ^‡^		4.46
Piperaquine	3.52	7.90 ^*^		2.24
Lumefantrine	3.93	13.8 ^*^		3.58
Artemether	3.28	33.4 ^€^		10.2
Chloroquine	4.47	27.0 ^€^		6.04
DEAQ	3.44	18.40 ^‡^		5.33

Using Student’s t- test the differences between the sensitive parental line and amodiaquine resistant line were significant ^*^ *p* < 0.01 ^‡^ *p* < 0.001 ^€^ *p*< 0.0001

### Amodiaquine resistance associated with cross-resistance to CQ, LM, PMQ, PQ and ATM

The selection of stable AQ resistant parasites allowed us to study whether AQ resistance also reduced the susceptibility of other antimalarial drugs ( Dataset 2). Using dilution cloned parasite, we determined the ED _90 _of PQ, LM, PMQ and ATM against both the AQ sensitive (AQS) and AQR. To our surprise, the AQR yielded moderate and slight resistance to ATM (I _90_ = 10.2) and PMQ (I _90_ = 5.8) respectively. Interestingly, the AQR had a lower resistance level to PQ (I _90_ = 2.2-fold) when compared with LM (I _90_ = 3.5-fold), despite PQ and AQ belonging to the same chemical class of 4-aminoquinoline and LM belonging to the different chemical class of the aryl-alcohols ( Table 3b). Our results mean that the AQR also acquired mechanisms that confer resistance to ATM, LM, PQ, PMQ and CQ. The cross-resistance profile is not surprising for drugs such as CQ and PQ, since they are quinoline-based compounds, and chemically related to AQ, thus may share some resistance mechanisms. Indeed, selection of the CQ resistance in *P. berghei* has previously been shown to confer cross-resistance to AQ, mefloquine and PMQ, two quinoline-based drugs ( Platel *et al.,* 1998). Similarly, we expect PMQ (8-amino quinoline) and LM (an aryl-alcohol) to share specific mechanisms with 4-amino quinoline-based on the similarity in the modes of action. However, the high cross-resistance levels for ATM (I _90_ = 10fold) is entirely surprising. Artemether is mechanistically and chemically unrelated to AQ ( Robert *et al.*, 2001; Tilley *et al.*, 2016). Amodiaquine inhibits heme polymerization within the digestive vacuole, thus killing the parasite by the accumulation of toxic heme ( O’Neill *et al.*, 2006). Artemisinins has multiple targets, for instance, the heme digestion pathway ( Klonis *et al.*, 2011), inhibition of the translationally controlled tumour protein ( *TCTP*) and the *PfATP6*, a sarcoplasmic-endoplasmic reticulum calcium ATPase (SERCA) ( Eckstein-Ludwig *et al.*, 2003; Krishna *et al.*, 2008). Recently, phosphatidylinositol-3-kinase was validated as an artemisinin target with high levels of its product phosphatidylinositol-3-phosphate associating with artemisinin resistance in *P. falciparum* ( Mbengue *et al.*, 2015) *.* Since the mechanisms of action and resistance of ATM are different from that of AQ, the cross-resistance between these two drugs may be due to the alteration of the mechanisms of drug transport, drug metabolism and drug accumulation within the cells. To date, the combination of ATM/LM (Coartem ^®^), dihydroartemisinin/PQ (Artekin ^®^) and ASN/AQ are the drugs of choice in many sub-Saharan African countries ( WHO, 2016). Assuming the mechanism of resistance between *P. falciparum* and *P. berghei* are similar, then our results would suggest that selection of AQ resistance, a component of Coarsucam™ would compromise the efficacy of Artekin ^® ^and Coartem ^®^. However, so far studies in *P. falciparum* do not indicate a correlation between the decrease in AQ and artemisinin activity ( Borrmann *et al.*, 2013; Nsobya *et al.*, 2010).

### Evaluation of point mutation in *Pbcrt*, *Pbmdr1, Pbubp1 and PbKelch13* ( Dataset 3)

To investigate the possible resistance mechanisms, we first interrogated for polymorphisms in two drug resistance transporters in the malaria parasite, *the Pbcrt* and *Pbmdr1.* The two transporters directly mediate and modulate susceptibility to quinoline-based drugs in *P. falciparum*. Our study focused on the whole coding regions *of* these two genes *.* To date, several studies have demonstrated the association between 4-amino-quinoline resistance and the mutations in *crt* gene, changes in expression profiles and copy number variation in *the mdr1* gene ( Borges *et al.*, 2011; Duraisingh & Cowman, 2005; Dhingra *et al.*, 2017). The single nucleotide polymorphism (SNP) in *Pfcrt* (codon 76) associates with CQ and AQ resistance in *P. falciparum* ( Ecker *et al.*, 2012; Fidock *et al.*, 2000; Ochong *et al.*, 2003). Studies in the rodent malaria *Plasmodium chabaudi*, however, found no association between *crt* and CQ resistance ( Afonso *et al.*, 2006; Hunt *et al.*, 2004), suggesting that other genes may mediate CQ and the 4-aminoquinoline resistance. Recent studies also identified potential *crt* background mutations; Ile356Thr and Asn326Ser that associate with artemisinin resistance ( Miotto *et al.*, 2015). In the present study, the nucleotide codons corresponding to amino acid position 76, 326 and 356 of the *PbCRT* protein were found not to harbour any mutation in AQ resistant line (compared to the sensitive line). However, we observed a substitution mutation (A -> C 284) in the nucleotide sequence of the AQR, that resulted in a His95Pro mutation in the *PbCRT* protein. The His95Pro mutation localises within the second transmembrane domain close to the food vacuole compartment suggesting that the mutation could play a role in drug transport. However, the functional role and biological consequence of His95Pro mutation in AQ resistance require further investigation. We then extended our study to the *mdr1* transporter. Mutations at positions 86, 184, 1034, 1042, and 1246 of the *Pfmdr1* mediate and modulate CQ, LM and mefloquine resistance ( Ecker *et al.*, 2012; Price *et al.*, 1999; Price *et al.*, 2004; Sisowath *et al.*, 2005). Similarly, our recent investigation using LM and PQ resistant *P. berghei* parasite found no polymorphisms in *crt* and *mdr1* genes ( Kiboi *et al.*, 2014). Sequencing of the whole coding region of the *mdr1* from AQR and the AQS did not reveal any sequence variation. The presence of a novel mutation (His95Pro) in the crt gene coupled by the absence of hitherto known mutations within the *crt* and *mdr1* genes suggest that the malaria parasite may develop resistance by the acquisition of mutation in other positions of the proteins. Indeed, the addition of C101F mutation in the crt gene of the CQ resistant P. falciparum conferred high resistance to PQ but generated a reciprocal susceptibility to AQ, quinine and ATM ( Dhingra *et al.*, 2017). The specific introduction of the His95Pro mutation using CRISPR/Cas9 approach would provide additional insights on the role of the mutation in mediating AQ resistance as well as the quinoline drugs.

The AQ resistant line had significantly reduced sensitivity to ATM with an ED _90_ of 33.4mg/kg compared with an ED _90_ of 3.28 mg/kg for AQ sensitive, translating to a 10-fold difference. Recent reports have validated *Kelch13* propeller domain, Met476Ile, Tyr493His, Arg539Thr, Ile543Thr and Cys580Tyr mutations as markers for artemisinin resistance ( Miotto *et al.*, 2015; Straimer *et al.*, 2015). We hypothesised that *PbKelch13* might possess SNPs, and thus mediate this cross-resistance. Our data showed no mutation in *the PbKelch13* domain, thus AQ and ATM resistance observed *in vivo* is not associated with SNPs in *the Kelch13* domain. We focused our study on *Kelch13.* However other genes such as *TCTP*, *SERCA* and *PI3P* that associate with artemisinins action or resistance in *P. falciparum* ( Eckstein-Ludwig *et al.*, 2003) may also associate with our selected AQ resistant line. As the index of resistance to ATM (I _90_ = 10.2) was double that of AQ (I _90_ = 4.2) indicate that AQ and ATM could share some resistance mechanisms in *P. berghei*. Thus, these AQ resistant lines could be used to define these shared mechanisms, and some of them may be *TCTP*, *SERCA* and *PI3P* or other unknown genes.

To further understand the AQ and ATM resistance in AQR, we focused on the *ubp1* gene. The acquisition of V739F and V770F mutations in *the* conserved C-terminal region of the *ubp1* is associated with artesunate resistance in *P. chabaudi* ( Hunt *et al.*, 2010). Similarly, Tyr835Ly and Ser836Gln mutations occurred in both LM and PQ resistant *P. berghei* (unpublished data: Kiboi, Irungu, Orwa, Kamau, Ochola-Oyier, Ng’ang’a and Nzila). In our current study, the analysis of the sequence fragments flanking 739, 770, 834 and 835 positions of the *PbUBP1* protein revealed no amino acid changes in the selected AQR. Studies in *P. falciparum in vitro* also found no association between artemisinin resistance and mutation in *ubp1* ( Chavchich *et al.*, 2010) *;* however, analysis of field *P. falciparum* isolates from Western Kenya associated *Pfubp1* Glu1528Asp mutation with tolerance to artemisinin ( Henriques *et al.*, 2015). We thus envisage complex mechanisms controlling loss of ATM efficacy in the AQ resistant phenotype. Examining the whole genome and transcriptome profile may expose these complex networks.

### High mRNA transcripts of *Pbmdr1*, *Pbnhe1*, *Pbvp2* and *Pbcvx1* associated with AQ resistance

**Figure 4.**
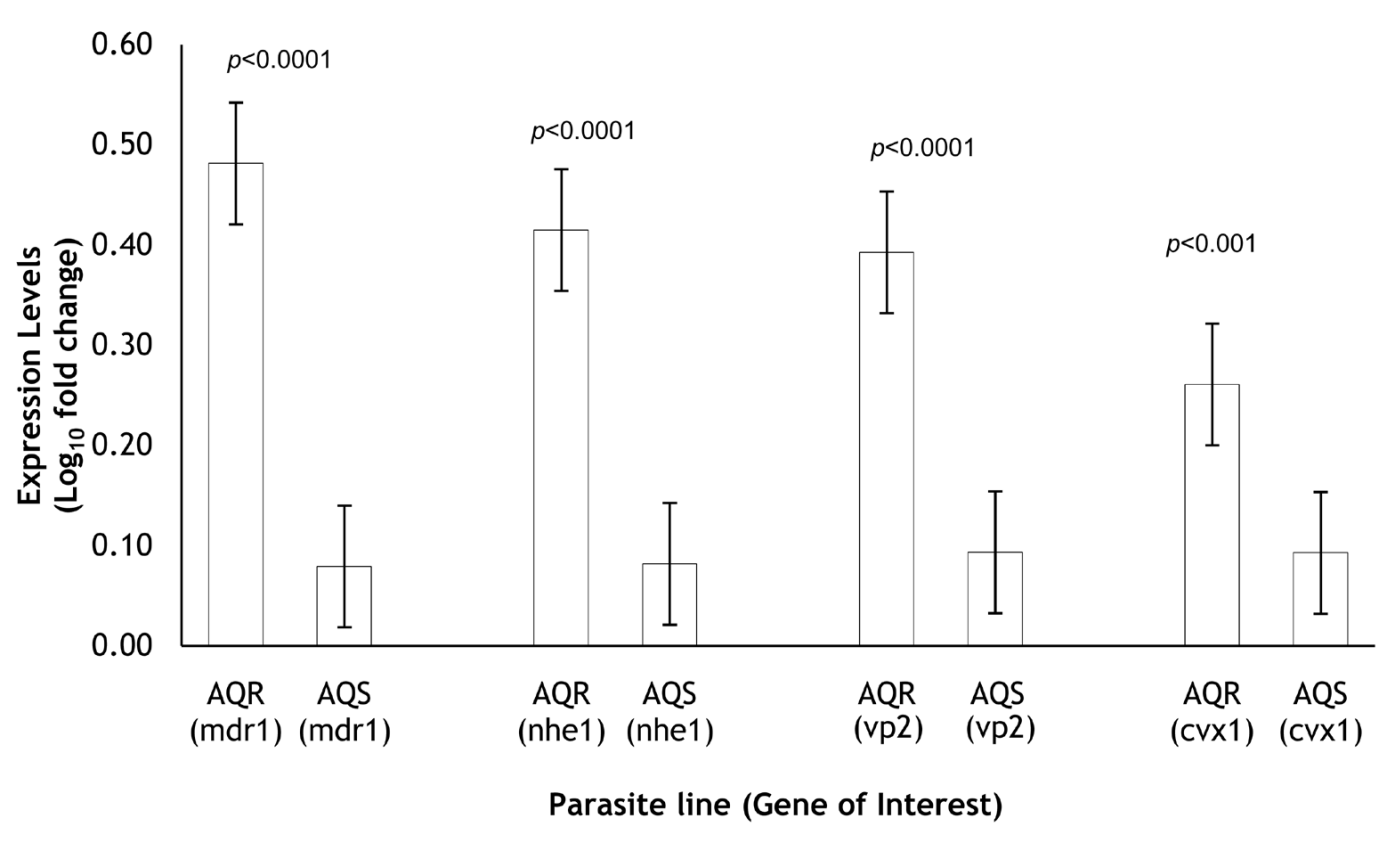
Expression profiles of target drug resistance genes. The multidrug resistance gene 1 ( *mdr1*), sodium hydrogen exchanger ( *nhe1*), V-type H+ pumping pyrophosphatase ( *vp2*) and Ca ^2+/^H ^+^ antiporter ( *vcx1*). Expression level was measured from cDNA amount derived from 5µg of total RNA isolated from amodiaquine resistant (AQR) relative to the wild-type amodiaquine sensitive (AQS) clones. The differential expression from a mean of three independent experiments and technical triplicates were significantly different for *mdr1* (p<0.0001), *nhe1* (p<0.0001), *vp2* ( *p<0.0001*) and *cvx1* ( *p<0.001*) after student’s *t-test* analysis with p-value set at 0.05.

To further probe other probable mechanisms of AQ resistance, we hypothesised that essential transporters or ion exchangers, *Pbmdr1*, *Pbnhe1*, *Pbvp2* and *Pbcvx1* could mediate AQ resistance via altered mRNA transcript levels ( Dataset 3). The results show that the mRNA transcript of *Pbmdr1* and *Pbvp2* were elevated 3.0fold ( *p<0.0001*) and 2.3fold ( *p<0.0001*), respectively ( Figure 4). Concerning the *Pbnhe1* and *Pbcvx1*, the AQR had a significantly high amount of *Pbnhe1* mRNA transcripts of 2.6fold compared to the AQS ( *p<0.0001*), and similar results were recorded on *Pbcvx1*, 1.7fold ( *p<0.001)* ( Figure 4). Therefore, high *mdr1*, *vp2, cvx1 and nhe1* transcript level associated with AQ resistance. The overexpression of *mdr1* is a marker for *P. falciparum* resistant to MQ, AQ, CQ and ATM ( Borges *et al.*, 2011; Gonzales *et al.*, 2008). However, the amplification of *mdr1* gene was not linked with CQ and PQ resistance in *P. falciparum* ( Sidhu *et al.*, 2006; Witkowski *et al.*, 2017), suggesting a complex regulation of the resistance mechanisms for the quinoline related drugs. Also, the *mdr1* regulates transcription of other drug resistance genes ( Gonzales *et al.*, 2008; Jiang *et al.*, 2008). For instance, augmenting CQ resistance in parasites harbouring *Pfcrt* K76T mutation ( Fidock *et al.*, 2000). Here, we show that *mdr1* overexpression may play a direct role in mediating AQ resistance.

Two genes, *vp2* and *cvx1*, are H ^+^ channel molecules that play two roles in CQ resistance: regulation of *pH* balance in the parasite's food vacuole and a compensatory role (adaptive changes in response to the mutation in drug resistance genes) in a mutated *Pfcrt* protein ( Jiang *et al.*, 2008). In a recent report, the PQ resistance was associated with a high *vp2* and *cvx1* expression in *P. berghei*, though there was no mutation in the *Pbcrt* gene ( Kiboi *et al.*, 2014). The AQ resistant line carried a His95Pro mutation in *PbCRT* protein. Thus, the elevation of *vp2* and *cvx1* may compensate for this mutation, as it has previously reported with the Lys76Thr crt mutation in *P. falciparum*. To date, the proffered mode of action for CQ, AQ and PQ is the inhibition of heme polymerisation within the food vacuole ( O’Neill *et al*., 2011). Based on this mode of action, some resistance mechanisms associated with AQ may involve proteins within the food vacuole. We thus argue that high *vp2* and *cvx1* expression may play a role in regulating pH balance in AQ resistance. Lastly, we report a 2.6-fold increase in *nhe1* mRNA transcript in AQ resistance in *P. berghei* ANKA. A report in *P. falciparum* has shown that quinine resistance can be associated with increased expression of *nhe1* in the presence of mutations in *Pfcrt* and *Pfmdr1* ( Nkrumah *et al.*, 2009) *.* Since the *nhe1* to regulates the Na ^+^ and H ^+^ exchange, this ion exchanger may also contribute to the resistance in AQR parasite lines.

In conclusion, we provide essential evidence about AQ resistance in *P. berghei* ANKA. First, the emergence of AQ resistance led to the loss of susceptibility to ATM, PMQ, LM, PQ and CQ; thus, the AQ resistant parasite is a "multi-drug" resistant parasite. Second, a novel His95Pro mutation in *PbCRT* is associated with AQ resistance and may well mediate the cross-resistance profiles. Third, one route for acquiring AQ resistance is via increased transcription of *mdr1*, *nhe1*, *vp2* and *cvx1 genes.* These genes augment the resistance levels and confer a physiological advantage to drug resistance genes that may possess biologically deleterious mutations ( Gonzales *et al.*, 2008). The elevated expression of these genes is consistent with *P. falciparum* resistance to CQ, LM and ATM ( Gonzales *et al.*, 2008; Jiang *et al.*, 2008; Mwai *et al.*, 2012), suggesting that some mechanisms between *P. falciparum* and *P. berghei* are similar. Finally, AQ resistance and its associated cross-resistance profiles are independent of SNPs in *ubp1* and *Kelch13* genes. Studies are underway to explore the whole genome to reveal other possible SNPs and copy number variants associated with AQ resistance.

## Data availability

The raw data for this study are deposited in OSF as follows:

**Dataset 1**: Parasite densities in the 4DT used for determination of 50% and 90% effective dose, https://doi.org/10.17605/OSF.IO/NWPXK ( Kiboi, 2018a).

**Dataset 2**: Parasite densities for cross resistance profiles, https://doi.org/10.17605/OSF.IO/KTSYB ( Kiboi, 2018b).

**Dataset 3**: Expression level profiles and sequence data of resistance genes, https://doi.org/10.17605/OSF.IO/VH9RY ( Kiboi, 2018c).
