# Supplementary material for: Amodiaquine resistance in Plasmodium berghei is associated with PbCRT His95Pro mutation, loss of chloroquine, artemisinin and primaquine sensitivity, and high transcript levels of key transporters
Source: Wellcome Open Res. 2018 Jun 6;2:44. Originally published 2017 Jun 20. [Version 2] doi: 10.12688/wellcomeopenres.11768.2 (PMC5998014; doi:10.12688/wellcomeopenres.11768.2)
Supplement: Peer review history

# Peer review: Referee response for version 2

**DOI:** 10.21956/wellcomeopenres.15937.r33267

**Published:** 2018-06-20

**Authors:** Richard T. Eastman

**Affiliations:** 1 National Center for Advancing Translational Sciences, National Institutes of Health, Bethesda, MD, USA

The authors have significantly improved the manuscript, and the additional data further supports their hypothesis that the observed mutation in PbCRT may modulate amodiaquine susceptibility.

 For the experiments assessing the stability of the amodiaquine resistance, both after freezing of the parasite line and passage in the absence of drug selection pressure, there is a notable reduction in the ED50 values, but maintenance of the ED90 levels. Is this difference significant (would be helpful for statistical analysis to be conducted for all comparisons). In addition, discussion (with possible inclusion of a dose response supplemental figure) would be helpful to understand this altered drug response phenotype (alteration in the slope of the amodiaquine dose response after removal of drug pressure). As this may suggest two independent genetic/epigenetic elements contributing to the observed amodiaquine resistance, where one is stable through the freezing/thawing process and serial passage without drug selection pressure and the other reverts without maintained selection pressure.

I have read this submission. I believe that I have an appropriate level of expertise to confirm that it is of an acceptable scientific standard.

# Peer review: Referee response for version 2

**DOI:** 10.21956/wellcomeopenres.15937.r33269

**Published:** 2018-06-13

**Authors:** Axel Martinelli

**Affiliations:** 1 Research Center for Zoonosis Control, Hokkaido University, Sapporo, Japan; 2 Biological and Environmental Sciences and Engineering (BESE) Division, King Abdullah University of Science and Technology (KAUST), Thuwal, Saudi Arabia

I am pleased to see that the authors addressed my main concern regarding the stability of the resistant phenotype by passaging the resistant line in the absence of drug selection and confirmed the acquisition of stable AQ resistance.

 There are still occasional small grammar mistakes in the main text, although I do take note of the fact that the written English has been considerably improved. I will let the editor decide whether this minor issue requires further action or not.

 I noticed the authors ordered their references alphabetically rather than chronologically (i.e. from oldest to most recent) when citing them in the main text. I am used to the latter format, but if the alphabetical format is according to the journal guidelines. I have no further comments.

 The His95Pro mutation may play a role, but until other mutations can be excluded by WGS and the role is verified by transfection studies, I would refrain from asserting that it is associated with AQ resistance (although it does make a plausible candidate). Thus I would change the concluding remark:

 "Second, a novel His95Pro mutation in *PbCRT* is associated with AQ resistance:

 to

 "Second, a novel His95Pro mutation in *PbCRT* may be associated with AQ resistance"

I have read this submission. I believe that I have an appropriate level of expertise to confirm that it is of an acceptable scientific standard.

# Peer review: Referee response for version 2

**DOI:** 10.21956/wellcomeopenres.15937.r33268

**Published:** 2018-06-11

**Authors:** David A. Fidock

**Affiliations:** 1 Department of Microbiology and Immunology, Columbia University Medical Center (CUMC), New York, NY, USA

I am satisfied with their revision and support the indexing of this report, which provides interesting data on an important topic in antimalarial chemotherapy

I have read this submission. I believe that I have an appropriate level of expertise to confirm that it is of an acceptable scientific standard.

# Peer review: Referee response for version 1

**DOI:** 10.21956/wellcomeopenres.12712.r25295

**Published:** 2017-09-07

**Authors:** Richard T. Eastman

**Affiliations:** 1 National Center for Advancing Translational Sciences, National Institutes of Health, Bethesda, MD, USA

The emergence and spread of drug resistance parasites remains a constant concern and threatens to reverse the reduction of malaria related morbidity and mortality. Amodiaquine is widely used to treat malaria episodes, combined with artesunate, and as a prophylactic, combined with sulfadoxine-pyrimethamine. As such the elucidation of genetic determinates underlying decreased drug susceptibility would permit genetic surveillance for the emergence and spread of amodiaquine-resistant parasites, facilitating adequate public health measures to assure proper utilization of antimalarial therapy. Ndung’u *et al.* report the *in vivo *drug selection and characterization of amodiaquine-resistant *Plasmodium berghei *parasites. This represents an essential first-step in the elucidation of genetic determinants underlying the resistance phenotype. As the authors note, further characterization of the lines (whole genome sequencing/RNA-seq/genetic backcross), along with validation studies will be required to further support this association based initial characterization.

  

1. From the Methods section, it is unclear if the *P. berghei* ANKA line was cloned prior to selection experiments. This would limit the impact that initial sub-populations contribute to the identified pre-selection/post-selection genetic variances. If the line wasn’t cloned prior to the selection this may partially explain the multiple genetic differences identified (Pbcrt SNP along with expression variance in four distinct transporters).
2. It is unclear from the Methods section if independent PCR reactions/sequencing of both strands were performed on the target loci indicated in Figure 1, to address polymerase/sequencing errors.
3. The depiction of the drug selection procedure in Figure 2 is confusing as the parasites were subjected to an increasing drug concentration selection (initial oral treatment of the AQ ED99 concentration and “increasing concentrations…based on parasite growth”), not two groups (either 2.5 or 5mg/kg/day) as indicated. The figure should be revised to indicate the selection concentration used for each passage and day 4 parasitemia. Also unclear from the methods/figure is the robustness of the selection for each passage. As three mice were inoculated per passage, was there any variance in the positivity/parasitemia of the mice upon selection pressure? If these lines/sub-passages are preserved they may represent an exciting tool to dissect the evolution of AQ resistance (in a similar manner that Hunt *et al.* discerned drug resistance in *P. chabaudi*).
4. The authors utilize a single parasite freeze/thaw to assess stability of the drug resistance phenotype. It is suggested that serial passage in naïve, non-drug treated mice is a more stringent evaluation of the resistance stability. Another method would be the passage of the line through the mosquito stage (which could also be run in parallel with genetic back-crossing of the line). The lower IC50 and IC90 values after the freeze/thaw suggest some instability in the drug-resistance phenotype.
5. As the drug dose response is sigmodal a non-linear regression analysis is usually preferred. Although a linear regression analysis would typically have good estimation of the IC50 value, due to the linear nature of the slope, there is disparity in calculation of the IC90 value using a linear regression analysis.

I have read this submission. I believe that I have an appropriate level of expertise to confirm that it is of an acceptable scientific standard, however I have significant reservations, as outlined above.

# Response

**Published:** 2018-05-23

**Authors:** Daniel Kiboi

**Affiliations:** Kenya Medical Research Institute, Kenya

**Author’s Response**

 1. The parent line used for AQ resistance was a clonal parasite (676m1cl1). We have added this statement in the Method section (Parasite, Host and Compounds subsection). After the selection of the resistant line, parasite lines were cloned by limiting dilution before PCR amplification and sequencing of the *Pbmdr1*, *Pbcrt*, *Pbubp1 *and *PbKelch13 genes.*

 2. We amplified independent amplicons and sequenced in both forward and reverse direction. To have repeated the experiment by PCR amplifying and sequencing the whole coding region for both *Pbcrt *and *Pbmdr1 genes*. To minimise the possible polymerase errors, we used a proof-reading polymerase (Phusion Flash High Fidelity PCR Master Mix, Thermo Fisher Scientific). We have added these statements in the methodology and results section

 3.We have used percentage parasitaemia recorded during the drug selection process to revise Figure 2. We have revised the Figure 2 to show the percentage parasitaemia during selection process relative to the increasing drug pressure dosage. In response to the second question on the parasitaemia variance during the selection process. We recorded variances in the parasitaemia between the three mice used for selecting AQ resistance at each of the drug pressure passage. Parasite lines for each mouse and at each drug pressure passage were cryopreserved. We agree with the reviewer that one robust way of dissecting the mechanisms of AQ resistance is to use the Linkage Group Selection (Culleton et al. 2005; Hunt et al. 2007; 2010) followed by whole genome and transcriptome sequencing. We hope to use these approaches in our current studies.

 4. We concur with the reviewers that performing drug-free passage is a more stringent approach for ascertaining the stability of the AQ resistant. We therefore evaluated and confirmed the stability of the AQ resistant parasite by culturing the resistant parasite for a total of ten passages in the absence of the AQ. We then determined the ED _50_ and ED _90_ using the standard 4DT test. Figure 3 illustrates this data on the stability of the mutant parasites. Table 3a also contains the new computed ED _90_ and indices of resistance.

 5.We consistently used the linear regression analysis in estimation of the ED50 and ED90. Since, the linear regression would provide a good estimate, we presume our results on the ED50 and ED90 would correlate well across the different assays we conducted.

# Peer review: Referee response for version 1

**DOI:** 10.21956/wellcomeopenres.12712.r23650

**Published:** 2017-07-17

**Authors:** David A. Fidock

**Affiliations:** 1 Department of Microbiology and Immunology, Columbia University Medical Center (CUMC), New York, NY, USA

This is an interesting report on an important topic. The authors have made an important contribution by selecting a rodent malaria parasite line (in *Plasmodium berghei*) that is resistant to amodiaquine (ADQ), an antimalarial combination therapy partner drug. The results implicate a novel mutation in the *Plasmodium berghei PbCRT* ortholog of the *Plasmodium falciparum* chloroquine resistance transporter *Pfcrt*. This P95H is associated with reported changes in parasite sensitivity to multiple antimalarials including ADQ and its metabolite monodesethyl-ADQ, as well as piperaquine, lumefantrine, artemether and chloroquine.

 This is a preliminary assessment of this drug-resistant parasite line. One important caveat is that the authors only targeted certain regions of *PbCRT *or the other likely resistance determinant *Pbmdr1* ( *P. berghei *multidrug resistance gene-1). Other mutations might therefore have appeared that were not detected. The authors have not performed gene editing to confirm whether or not the *PbCRT* H95P mutation can account for the full extent of altered antimalarial susceptibilities that were observed in their mutant line.

 To provide a comprehensive assessment of the genetic basis of resistance, the authors should perform whole-genome sequence analysis of the mutant compared to the parental line. If that is not feasible, the authors should at the very least complete their sequencing of the entire coding sequence for both *PbCRT *and *Pbmdr1*. For *PbCRT*, full-length sequences can be obtained from reverse-transcribed cDNA (they already report making RNA for some of their qRT-PCR studies).

 Other points:

1. Introduction: It is not entirely correct that selected resistant parasites in *P. falciparum* are generally not stable. This argument should be removed.
2. Figure 2 should show what regions were adequately sequenced.
3. Concluding that the lines have a stable resistant phenotype after being stored at -80°C for one month is an overstretch. Stability usually means that the phenotype persists for one month or more of continuous propagation without drug pressure. Especially as the ED _50_ and I _50_ values post-thawing are ~1/2 that of the pre-freezing line on passage #36 (see Table 3A). The authors should either test for true stability in the absence of drug, or remove this as a central finding of their study.
4. Table 3B – the authors cannot base any changes in mutant parasite susceptibility to primaquine or lumefantrine based on a comparison with earlier published data for those drugs with the sensitive parental line. If the parent was not tested here in parallel with the mutant for these drugs, then those data should be removed, or at the very least they should attenuate their statements and list the caveat that data for the parental line were from separate studies and thus shifts in susceptibility have not been directly demonstrated. Also for Table 3, the authors need to list how the number of independent experiments and mouse group sizes.
5. On page 10 the authors state that *Pfmdr1 *overepression is a common marker of resistance to chloroquine. Results presented in Sidhu *et al* 2007 J Infect Dis showed no change in chloroquine IC _50_ in isogenic lines with different *Pfmdr1* copy numbers and that work should be cited.
6. Figure 2 is hard to understand as it seems to indicate that selection was only performed at two fixed concentrations, whereas resistance was obtained using a ramping procedure. The authors should clarify what is being shown.
7. Figure 3 is also non-intuitive. Are the data shown relative to a reference gene? This should be listed in the legend. Were these three independently prepared and harvested cultures? Or are these technical triplicates form the same set of cultures?    

I have read this submission. I believe that I have an appropriate level of expertise to confirm that it is of an acceptable scientific standard, however I have significant reservations, as outlined above.

# Response

**Published:** 2018-05-23

**Authors:** Daniel Kiboi

**Affiliations:** Kenya Medical Research Institute, Kenya

We concur that performing whole genome sequencing of the resistant line may comprehensively dissect AQ resistance markers. This objective is in our current study plan; however, we have sequenced full-length of *the Pbcrt *and *Pbmdr1* genes from cDNA. We have edited the methods section to highlight the sequencing approach and included this data in the results section. We have also revised the Figure 1 to portray the sequenced region of the other two genes; Pbubp1 and Pbkelch13. We have included the new sequence data in dataset 3.

Response to other comments

1. We have removed the statement on "that selected resistant parasites in *P. falciparum* are generally not stable."

2. We have edited the Figure 1 to portray the  regions of the genes that were adequately sequenced 

3. We tested the stability of the AQ resistant parasite by culturing the resistant parasite for a total of ten passages in the absence of the AQ. We then determined the 50% and 90% effective dosages (ED _50_ and ED _90_) using the standard 4DT test.  In the MATERIALS AND METHODS ****section, we have included a statement on the stability assays, under the subsection “Submission of the parasite to AQ pressure and Resistance Level Test.  In the RESULTS AND DISCUSSION section, we have included the ED _50_ and ED _90 _values in Table 3a to illustrate the stability of the AQ-resistant parasites. We have included Figure 3 to show the drug response profile of the AQR, drug-free parasite and drug sensitive parent line. This data is under subsection on “Amodiaquine drug pressure induces stable-resistant phenotypes” We have included a new figure (Figure 3) in our revised version.

4. We have retested the ED _90_ for primaquine and lumefantrine against the sensitive parent parasite. We have included the new data on ED _90_ in Table 3b and the raw data in dataset 2.  We used at least four different drug concentrations and at least four Swiss mice per drug concentration. We have clarified this statement in Table 3a.

5. We have included the statement on the lack of association between CQ resistance and amplification of the *Pfmdr1* gene. We have cited Sidhu et al. 2007 study.

6. Using data from drug pressure and at different passage stages, we have revised the Figure 2 to show the percentage parasitaemia during selection process relative to the increasing drug pressure dosage.

7. We have included the reference gene used to normalise the expression level data. We used technical triplicates from three independently prepared cultures. We have clarified this statement in the figure legend. Since we added a new figure on the stability of the amodiaquine resistant parasite, Figure 3 in our first version changes to Figure 4.

# Peer review: Referee response for version 1

**DOI:** 10.21956/wellcomeopenres.12712.r23654

**Published:** 2017-07-12

**Authors:** Axel Martinelli

**Affiliations:** 1 Research Center for Zoonosis Control, Hokkaido University, Sapporo, Japan; 2 Biological and Environmental Sciences and Engineering (BESE) Division, King Abdullah University of Science and Technology (KAUST), Thuwal, Saudi Arabia

The article presents the selection of a line of the rodent malaria parasite *Plasmodium berghei *for amodiaquine (AQ) resistance. This is potentially interesting work, due to the primary role played by AQ as a partner drug in artemisinin combination therapy (ACT).

 There are however two main issues that the authors must address before the paper can be accepted for publication.

 The first is unfortunately experimental. Persistence of drug resistant phenotypes can be unstable in malaria parasites and testing it after recovering parasites from deep freeze is not enough to guarantee that the phenotype is due to mutations rather than transient epigenetic effects. The fact that the ED _50_ doses are half or less than those measured before deep freezing further emphasises this concern.

 A far better way to ensure stability of the phenotype is either through passaging the resistant line for several rounds in mice in the absence of any drug pressure and/or passaging the resistant line through mosquitoes (e.g. Hayton *et al*, 2002; Afonso *et al* 2006; Kiboi *et al*, 2009). After the passaging protocol has been satisfied, the line can be tested for drug resistance.

 I urge the authors to perform this test. I do realise that this step will take a couple of months, but I am afraid it is necessary in order to ensure the stability of the phenotype.

 The second main issue is the quality of the written English. The manuscript is peppered with grammatical and style errors. This results in sometimes confusing and awkward sentences that affect a proper review of the content. The authors should consider rewriting the manuscript with the help of a native English speaker to ensure it meets the standards required for a scientific publication.

 A minor issue is that references should be ordered chronologically in the main manuscript when used together (e.g. Duraisingh and Cowman, 2005; Holmgren *et al*, 2006; Borges *et al*, 2011). At the moment the order appears to be rather random.

 I believe that selecting lines of malaria parasites for drug resistance to understand its genetic basis is essential to provide effective therapies for the treatment of this disease. Thus the work presented here is of interest to the scientific community, but only if the authors address the aforementioned issues.

 I also understand that the authors are in the process of sequencing the whole genome (and I presume transcriptome) of their AQ resistant line and it will be interesting to see what mutations may have arisen. If the line is indeed phenotypically stable, the authors should consider crossing it with a genetically distinct susceptible strain and then apply Linkage Group Selection (Culleton *et al*, 2005) to identify mutations underlying the phenotype. I could provide more details about how to proceed, should the authors decide to do so.

I have read this submission. I believe that I have an appropriate level of expertise to confirm that it is of an acceptable scientific standard, however I have significant reservations, as outlined above.

## References

[1] HaytonKRanford-CartwrightLCWallikerD: Sulfadoxine-pyrimethamine resistance in the rodent malaria parasite Plasmodium chabaudi. *Antimicrob Agents Chemother*.2002;46(8) :2482-9 1212192210.1128/AAC.46.8.2482-2489.2002PMC127344

[2] AfonsoAHuntPCheesmanSAlvesACCunhaCVdo RosárioVCravoP: Malaria parasites can develop stable resistance to artemisinin but lack mutations in candidate genes atp6 (encoding the sarcoplasmic and endoplasmic reticulum Ca2+ ATPase), tctp, mdr1, and cg10. *Antimicrob Agents Chemother*.2006;50(2) : 10.1128/AAC.50.2.480-489.2006 480-9 10.1128/AAC.50.2.480-489.2006 16436700PMC1366921

[3] KiboiDMIrunguBNLangatBWittlinSBrunRCholletJAbiodunONgangaJKNyambatiVCRukungaGMBellANzilaA: Plasmodium berghei ANKA: selection of resistance to piperaquine and lumefantrine in a mouse model. *Exp Parasitol*.2009;122(3) : 10.1016/j.exppara.2009.03.010 196-202 10.1016/j.exppara.2009.03.010 19318094PMC2691925

[4] CulletonRMartinelliAHuntPCarterR: Linkage group selection: rapid gene discovery in malaria parasites. *Genome Res*.2005;15(1) : 10.1101/gr.2866205 92-7 10.1101/gr.2866205 15632093PMC540282

# Response

**Published:** 2017-07-17

**Authors:** Daniel Kiboi

**Affiliations:** Kenya Medical Research Institute, Kenya

We thank the referee for insightful comments. 

To address the experimental concern raised, we are currently carrying out further experimentation on stability test. We choose to pass the parasite line through mice for at least five- ten drug free passages. We will then determine the resistance level after the drug free passages.

We recognize that passing the resistant line through mosquitoes as one of the ways of verifying stability of the phenotype. Since this is an ongoing project, we hope to use this approach as well before sequencing the genome and the transcriptome of the resistant line.

To address the second major concern, we have requested a native English speaker to assist in rewriting and improving the manuscript to the required publication standards.

# Response

**Published:** 2018-05-23

**Authors:** Daniel Kiboi

**Affiliations:** Kenya Medical Research Institute, Kenya

1. We tested the stability of the AQ resistant parasite by culturing the resistant parasite for a total of ten passages in the absence of the AQ. We then determined the 50% and 90% effective dosages (ED _50_ and ED _90_) using the standard 4DT test. In the materials and methods ****section, we have included a statement on the stability assays, under the subsection “Submission of the parasite to AQ pressure and Resistance Level Test. In the results and discussion section, we have included the ED _50_ and ED _90 _values in Table 3a to illustrate the stability of the AQ-resistant parasites. We have included a new figure (Figure 3) to show the drug response profile of the AQR, drug-free parasite and drug sensitive parent line. This data is under subsection on “Amodiaquine drug pressure induces stable-resistant phenotypes. We have uploaded the new data on stability in dataset 1.

2. We have improved the quality of the written English

3. We have corrected the order of the references

4. We plan to get in touch in with the reviewer as we embark to dissect further the molecular signatures associated with AQ resistance through sequencing the whole genome and transcriptome of the resistant parasites.
